# Supplementary material for: Measurement properties of outcome measures used in neurological telerehabilitation: A systematic review protocol
Source: PLoS One. 2022 Mar 21;17(3):e0265841. doi: 10.1371/journal.pone.0265841 (PMC8936471; doi:10.1371/journal.pone.0265841)
Supplement: S1 File — (DOCX) [file pone.0265841.s002.docx]

**Search Strategy**

| 1 | Nervous System Diseases.mp. or Nervous System Diseases/ |
| --- | --- |
| 2 | Brain disease.mp. or Brain Diseases/ |
| 3 | Traumatic brain injury.mp. or Brain Injuries, Traumatic/ |
| 4 | Cerebrovascular disorder.mp. or Cerebrovascular Disorders/ |
| 5 | Stroke/ or Stroke.mp. |
| 6 | Basal ganglia disease.mp. or Basal Ganglia Diseases/ |
| 7 | Parkinson.mp. or Parkinson Disease/ |
| 8 | Dystonia.mp. or Dystonia/ |
| 9 | Cerebellar disease.mp. or Cerebellar Diseases/ |
| 10 | Ataxia.mp. or Ataxia/ |
| 11 | Multiple sclerosis.mp. or Multiple Sclerosis/ |
| 12 | Spinal cord injury.mp. or Spinal Cord Injuries/ |
| 13 | Myelitis, Transverse/ or Myelitis/ or Myelitis.mp. |
| 14 | Poliomyelitis.mp. or Poliomyelitis/ |
| 15 | Peripheral nerve injury.mp. or Peripheral Nerve Injuries/ |
| 16 | Neuromuscular disease.mp. or Neuromuscular Diseases/ |
| 17 | Alzheimer Disease/ or Alzheimer.mp. |
| 18 | Movement disorder.mp. or Movement Disorders/ |
| 19 | Hemorrhagic Stroke/ or Stroke/ or Stroke.mp. or Ischemic Stroke/ |
| 20 | Telerehabilitation.mp. or Telemedicine/ or Telerehabilitation/ or Remote Consultation/ or Home Care Services/ |
| 21 | Telemetry.mp. or Telemetry/ |
| 22 | Video.mp. |
| 23 | Remote Sensing Technology.mp. or Remote Sensing Technology/ |
| 24 | Text Messaging.mp. or Internet/ or Text Messaging/ or Cell Phone/ |
| 25 | mobile health.mp. |
| 26 | tele health.mp. |
| 27 | Mobile Applications/ or mobile.mp. |
| 28 | computer.mp. or Computers/ |
| 29 | email.mp. or Electronic Mail/ |
| 30 | Virtual Reality/ or virtual.mp. |
| 31 | telecommunication.mp. or Telecommunications/ |
| 32 | remotely.mp. |
| 33 | telephone.mp. or Telephone/ |
| 34 | social media.mp. or Social Media/ |
| 35 | smartphone.mp. or Cell Phone/ or Computers, Handheld/ or Smartphone/ or Internet/ |
| 36 | Web Browser/ or web.mp. |
| 37 | Agreement.mp. |
| 38 | Psychometrics/ or "Reproducibility of Results"/ or Consistency.mp. |
| 39 | Internal consistency.mp. |
| 40 | Validity.mp. |
| 41 | Reliability.mp. |
| 42 | Ceiling effect.mp. |
| 43 | Floor effect.mp. |
| 44 | Sensitivity.mp. or "Sensitivity and Specificity"/ |
| 45 | Accuracy.mp. or Data Accuracy/ |
| 46 | "Reproducibility of Results"/ or Reproducibility.mp. |
| 47 | Repeatability.mp. |
| 48 | Applicability.mp. |
| 49 | Responsiveness.mp. or Psychometrics/ |
| 50 | Psychometric properties.mp. |
| 51 | Clinimetric properties.mp. |
| 52 | Psychometric data.mp. |
| 53 | Instrument psychometrics.mp. |
| 54 | Psychometric tests.mp. |
| 55 | Change score.mp. |
| 56 | Difference score.mp. |
| 57 | Generalizability.mp. |
| 58 | Minimal clinically important difference.mp. or Minimal Clinically Important Difference/ |
| 59 | MCID.mp. |
| 60 | Feasibility Studies/ or Feasibility.mp. |
| 61 | 37 or 38 or 39 or 40 or 41 or 42 or 43 or 44 or 45 or 46 or 47 or 48 or 49 or 50 or 51 or 52 or 53 or 54 or 55 or 56 or 57 or 58 or 59 or 60 |
| 62 | 1 or 2 or 3 or 4 or 5 or 6 or 7 or 8 or 9 or 10 or 11 or 12 or 13 or 14 or 15 or 16 or 17 or 18 or 19 |
| 63 | 20 or 21 or 22 or 23 or 24 or 25 or 26 or 27 or 28 or 29 or 30 or 31 or 32 or 33 or 34 or 35 or 36 |
| 64 | 61 and 62 and 63 |
